# Origin recognition complex subunit 1(ORC1) is a potential biomarker and therapeutic target in cancer

**DOI:** 10.1186/s12920-023-01691-9

**Published:** 2023-10-13

**Authors:** Linling Wu, Hui Chen, Chao Yang

**Affiliations:** 1 Integrated Chinese & Western Medicine Oncology Research Center, Jiangxi University of Traditional Chinese Medicine, Nanchang, 330004 China; 2 College of life science, Gannan Normal University, Ganzhou, 341000 China

**Keywords:** ORC1, Pan-cancer, Prognosis, Immune cells, Co-expression network

## Abstract

**Background:**

The origin recognition complex 1 (ORC1) is a large subunit of the origin recognition complex and acts as the master subunit of the precoding complex.

**Objective:**

To explore potential function and clinical significance of ORC1 in cancers.

**Methods:**

The expression level of ORC1 in different types of tumor tissues and matched normal tissues were detected by The Cancer Genome Atlas (TCGA) and validated by datasets from the gene expression omnibus (GEO) database. The association between ORC1 expression and infiltration levels of immune cell was analyzed. ORC1 and its co-expression genes were subjected to enrichment analysis to explore potential mechanisms in cancers, and the protein-protein interaction (PPI) network was constructed. Finally, the expression of ORC1 in tumor tissue and adjacent tissue was verified by immunohistochemistry (IHC).

**Results:**

ORC1 was highly expressed in the majority of tumors, and the expression level of ORC1 was associated with the pathological stages of ACC, LUAD, OV and SKCM. ORC1 was closely related with poor prognosis in ACC, LIHC, PAAD, READ and THCA. ORC1 in ACC and KICH was positively correlated with the infiltration level of immune cells while it was negatively correlated with the infiltration level of immune cells in THYM. Co-expression network analysis showed that CDCA3, GSG2, KIF2C, NCAPH and PLK1 were positively correlated with ORC1 in cancer, and enrichment analysis showed a correlation with cytosol, ATP binding and cell division. The expression of ORC1 in UCEC and KICH was higher than that in the adjacent tissues.

**Conclusion:**

ORC1 over-expressed in most tumors and could be severed as a novel biomarker for diagnosis. This study revealed that ORC1 might inhibit tumor immunity and might be a potential therapeutic target in cancers.

**Supplementary Information:**

The online version contains supplementary material available at 10.1186/s12920-023-01691-9.

## Introduction

With the continuous development of society, the pollution of living environment is becoming more and more serious, and people are coming into closer and closer contact with carcinogenic factors. The incidence of malignant tumors is increasing year by year, and malignant tumors have become the greatest enemy of human health. Although some progress had been made in targeted therapy, it is not enough [1]. In recent years, it had an increasing number of studies on tumors, but most studies have focused on tumour biomarker of individual tumors, so searching for pan-cancer biomarker is a common expectation [2, 3]. Meanwhile, the prognosis of tumors is poor due to the lack of early diagnostic indicators. In the current an important factor of poor response to cancer treatment is the failure to diagnose the disease early enough. There is an urgent need for bioinformatics techniques to find biomarker for early diagnosis of pan-cancer.

ORC is a protein complex containing 6 highly conserved subunits which is necessary to initiate DNA replication in eukaryotic cells [4]. ORC consists of ORC1 to 6, in which ORC1 is the largest subunit required to initiate DNA replication [5]. It is ubiquitous at the beginning of the S phase and is degraded, then re-synthesized at the end of the G2 phase and binds to chromosomes as the cell enters mitosis [6]. ORC1, also known as HSORC1 in humans, is poorly expressed in resting cells but can be up-regulated by cell growth signals [7].

In this study, we are the first using bioinformatics to analyze the relationship between ORC1 and pan-carcinogenesis. Meanwhile, the molecular mechanism of ORC1 in the occurrence and development of cancer was also discussed in multiple aspects. It was concluded that there were differences between ORC1 and prognosis in different tumor patients, and the relationship between cancer-related fibroblast infiltration. These results provide direction for the future research of ORC1.

## Materials & methods

### Differential expression analysis

GEPIA2 (http://gepia2.cancer-pku.cn), a website developed by Zhang Zemin’s laboratory at Peking University, was able to analyze the RNA-seq expression data of 9,736 tumor samples and 8,587 normal samples from the TCGA and GTEx projects [8]. Landing this web, we choose Box Plot of the Expression DIY, select missing tumor and normal samples in the TIMER, such as Acute Myeloid Leukemia (LAML), Brain Lower Grade Glioma (LGG), Testicular Germ Cell Tumors (TGCT) and so on, then plot the datasets. In the meantime, we can click the Stage Plot of Expression DIY, inputting the gene and choose some tumors we want to research. Next, we click the Survival Analysis and Similar Genes Detection, input the gene of ORC1,we can obtain these datasets. UALCAN (https://ualcan.path.uab.edu/) is an effective website for online analysis and mining of cancer data, mainly based on the relevant cancer data in TCGA database [9]. It can help medical personnel to identify and analyze the relevant genes by biomarker, survival analysis and so on, you can also query related information in other databases through related links. All in all, it is a simple, fast and effective TCGA data mining and analysis of the site tool. Clicking the CPTAC analysis of this web and input ORC1 in the search box, we can obtain the datasets by selecting Total-Protein and Phospho- Protein.

### Gene mutation analysis

cBioPortal (https://www.cbioportal.org/) is an open source resource for the interactive exploration of Multiple Cancer Genomics datasets [10, 11]. cBioPortal significantly reduces barriers to access between complex genomic data and cancer researchers, facilitating rapid, intuitive, and high-quality access to molecular profiling and clinical prognostic relevance for large-scale cancer genomics projects, and enable researchers to translate these data sets into biological insights and clinical applications. Inputting ORC1 in the quick search box, then we can get the datasets of the gene mutation in thirty tumors; clicking the mutation button, we can see the mutation locus of ORC1, and obtain the 3D view structure. In addition, the figures regarding the immunohistochemistry of ORC1 expression in human breast cancer, lung cancer, liver cancer, colon cancer tissues and normal tissues were obtained from the Human Protein Atlas (https://www.proteinatlas.org/).

### Immune infiltration analysis

The TIMER database (https://cistrome.shinyapps.io/timer/) was used RNA-Seq expression profiling data to detect the infiltration of immune cells in tumor tissue [12, 13]. The infiltration of six kinds of immune cells (b cells, CD4^+^ t cells, CD8^+^ t cells, Neutrphils, Macrophages and Dendritic cells) was studied. Landing this web, we input ORC1L (one nickname of ORC1) in the search box, and click the submit button. We also research the potential relationship between the level of infiltration of different immune cells and the expression of ORC1 gene in different types of TCGA, first click the Gene button of the Immune, input ORC1 in the Gene Expression and choose the cancer associated fibroblast as the Immune infiltrates. Then we use the Gene-Corr of the Expression to explore the correlation between five similar genes from GEPIA2 and ORC1 in varies tumor samples.

### Identification and enrichment analysis of co-expression genes

STRING (https://cn.string-db.org/) is a gene-protein interaction retrieval tool that enables users to easily access unique, wide-ranging experiments and predictive interaction information [14]. The interaction provided by string is based primarily on a confidence score, along with other ancillary information, such as protein domains and 3D structure. In this web, we input ORC1 in the protein name box and choose Homo sapiens as the datasets’ organism; in the settings, we make some changes of parameters: active interaction sources (experiments), minimum required interaction score [no confidence (0.150)], max number of interactors to show (no more than 50 interactors in 1st shell).

### Immunohistochemical

The chromophobe renal cell carcinoma tissue microarray ZL-KICC1601 and endometrial cancer tissue microarray ZL-UteS961 were purchased by Shanghai Wellbio technology Co.,Ltd. The ORC1 (F-10): SC-398,734 antibody was purchased from Santa Cruz Biotechnology. The chips were routinely dewaxed to water, incubated for 5 min with Ultra V Block, incubated for 2 h with ORC1 antibody working fluid 37℃, incubated for 20 min with enhancer, incubated for 30 min with secondary antibody after washing, rinsed and stained, sealing. The results were are analyzed by Quant Center2.3.

### Statistical analysis

Student’s t-test and one-way analysis of variance were applied for the statistical analysis. *P* < 0.05 indicated that there was a statistically significant difference.

## Results

### The expression of ORC1 in cancers

**Fig. 1:**
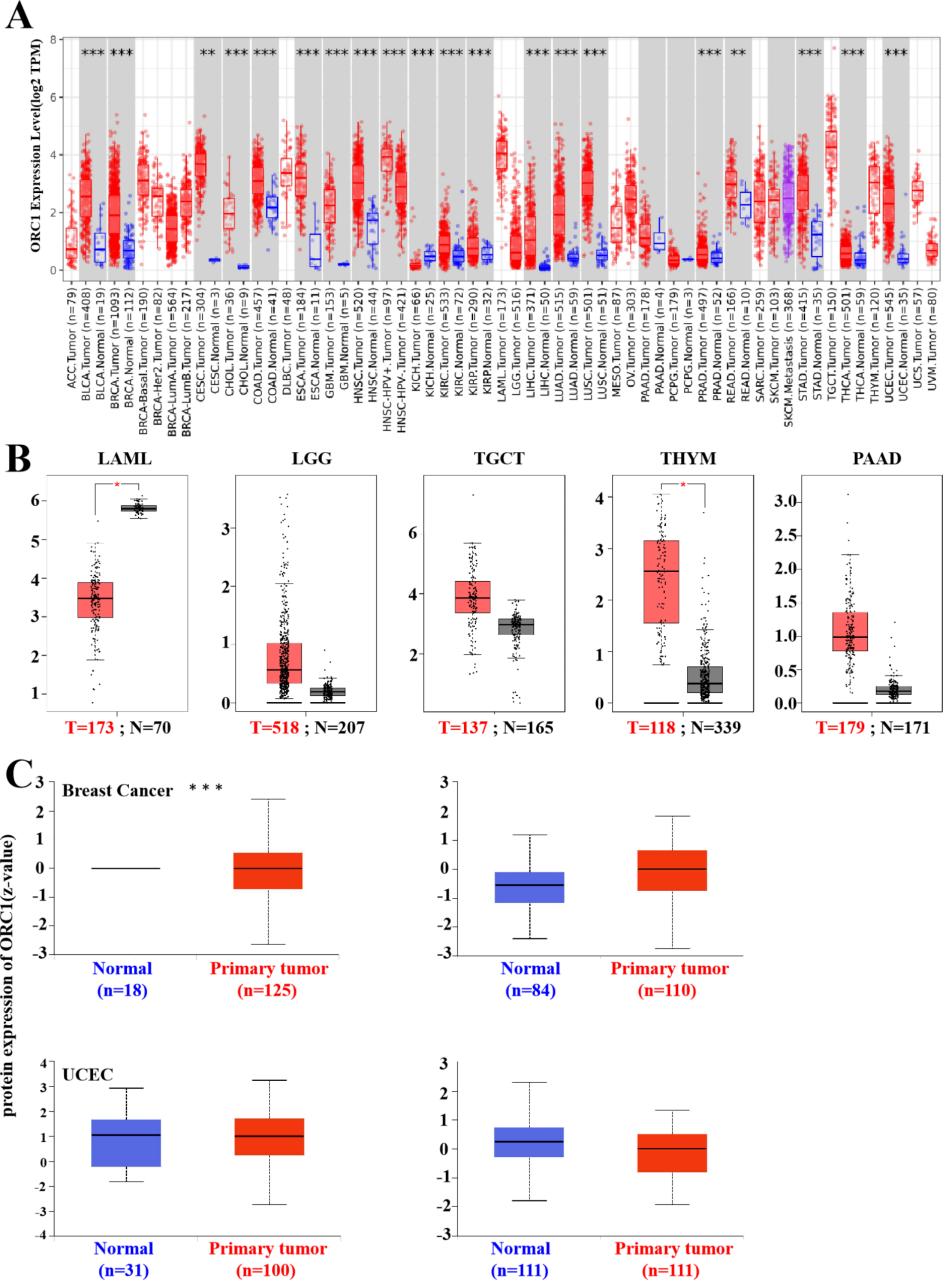
The relationship of ORC1 and human tumors (T = Tumor tissues, N = Normal tissues, n = the number of cases). **A**: Differential expression of ORC1 in different cancer or specific cancer subtypes tissues. **B**: Supplementary information of the missing message of Fig. 1A, such as LAML, LGG, TGCT, THYM and PAAD. **C**: Protein expression of ORC1 in Breast cancer, clear cell RCC, UCEC and lung adenocarcinoma

The expression level of ORC1 in different kinds of tumors and matched normal tissues were analyzed by TIMER, GEPIA2 and UALCAN. Figure 1A showed that the expression of ORC1 in most tumor tissue was higher than that of the normal tissue, such as Bladder Urothelial Carcinoma (BLCA) (T = 408, N = 19), Breast invasive carcinoma (BRCA) (T = 1093, N = 112), Colon adenocarcinoma (COAD) (T = 457, N = 41) and so on. The expression of ORC1 was the highest in testic tissue, followed by blood and Squamous cell of head and neck. Figure 1B is the supplementary data of Fig. 1A, including LAML (T = 70, N = 173), LGG (T = 518, N = 207), TGCT (T = 137, N = 165), Thymoma (THYM) (T = 118, N = 339), Pancreatic Adenocarcinoma (PAAD) (T = 179, N = 171). The data further supported that ORC1 expression in tumor tissue was significantly higher than that in normal tissue.

Then we studied the ORC1 protein levels in different tumors in UALCAN database (Fig. 1C), the available tumor data were BRCA, Kidney renal clear cell carcinoma (KIRC), Uterine Corpus Endometrial Carcinoma (UCEC), and Lung adenocarcinoma (LUAD). It was found that the protein expression level of ORC1 was significantly different between BRCA and normal tissue (P < 1E-12). Meanwhile, we can see the protein expression of ORC1 in KIRC tissue is higher than normal tissue. However, the expression of ORC1 in UCEC and LUAD are lower than corresponding normal tissues.

### Analysis of clinical parameters

**Fig. 2:**
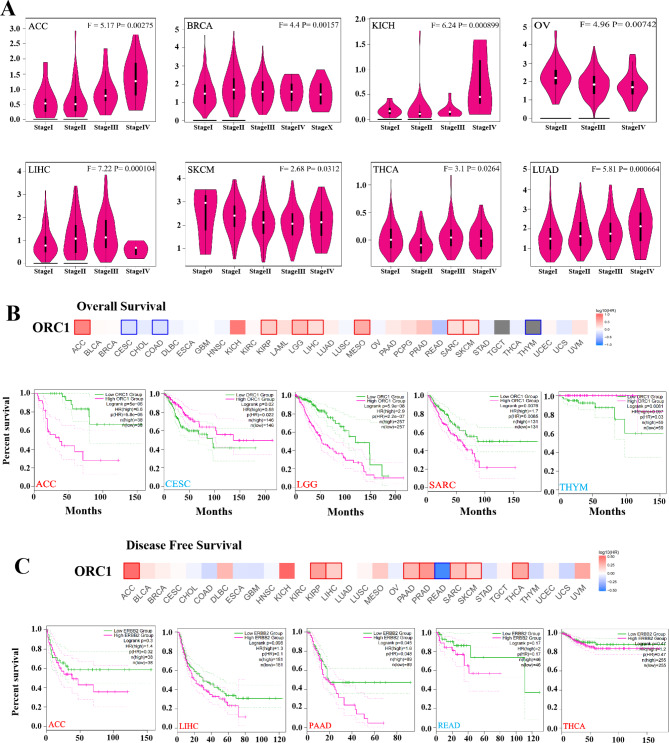
Expression of ORC1 in different clinical parameters. **A**: The pathological staging of ORC1 in ACC, BRCA, KICH, OV, LIHC, SKCM, THCA and LUAD [F = Fvalue, P = Pr(> F)]. **B**: The overall survival curve of ORC1 in ACC, CESC, LGG, SARC, THYM. **C**: The disease free survival curve of ORC1 in ACC, LIHC, PAAD, READ, THCA.

We analyzed the expression of ORC1 at different stages of the tumors by using Pathological Stage Plot module of GEPIA2 (Fig. 2A). The expression of ORC1 increased with the progression of the tumors in Adrenocortical carcinoma (ACC) and LUAD, while the expression of ORC1 decreased with the progression of the tumors in OV and Skin Cutaneous Melanoma (SKCM). In order to explore whether the expression of ORC1 can be used as a prognostic indicator of tumor patients, we analyzed the relationship between the expression level of ORC1 and the overall survival of tumors. According to the expression level of ORC1, we divided the tumor patients into high-expression Group and low-expression Group, and used TCGA and GEO datasets to study the correlation between ORC1 expression and prognosis of different tumor patients. The results showed that in ACC, LGG and Sarcoma (SARC), the overall survival rate of high-expression Group was significantly lower than that of low-expression Group. In CESC and THYM, low expression of ORC1 is associated with poor prognosis in overall survival (Fig. 2B). Figure 2 C showed that the disease free survival rate of low-expression Group was significantly higher in ACC, Liver hepatocellular carcinoma (LIHC), PAAD, Rectum adenocarcinoma (READ) and THCA, indicating that highly expressed ORC1 was closely related with poor prognosis in these tumors. In conclusion, the expression of ORC1 is differentially correlated with the prognosis in tumors.

### Gene mutation analysis

**Fig. 3:**
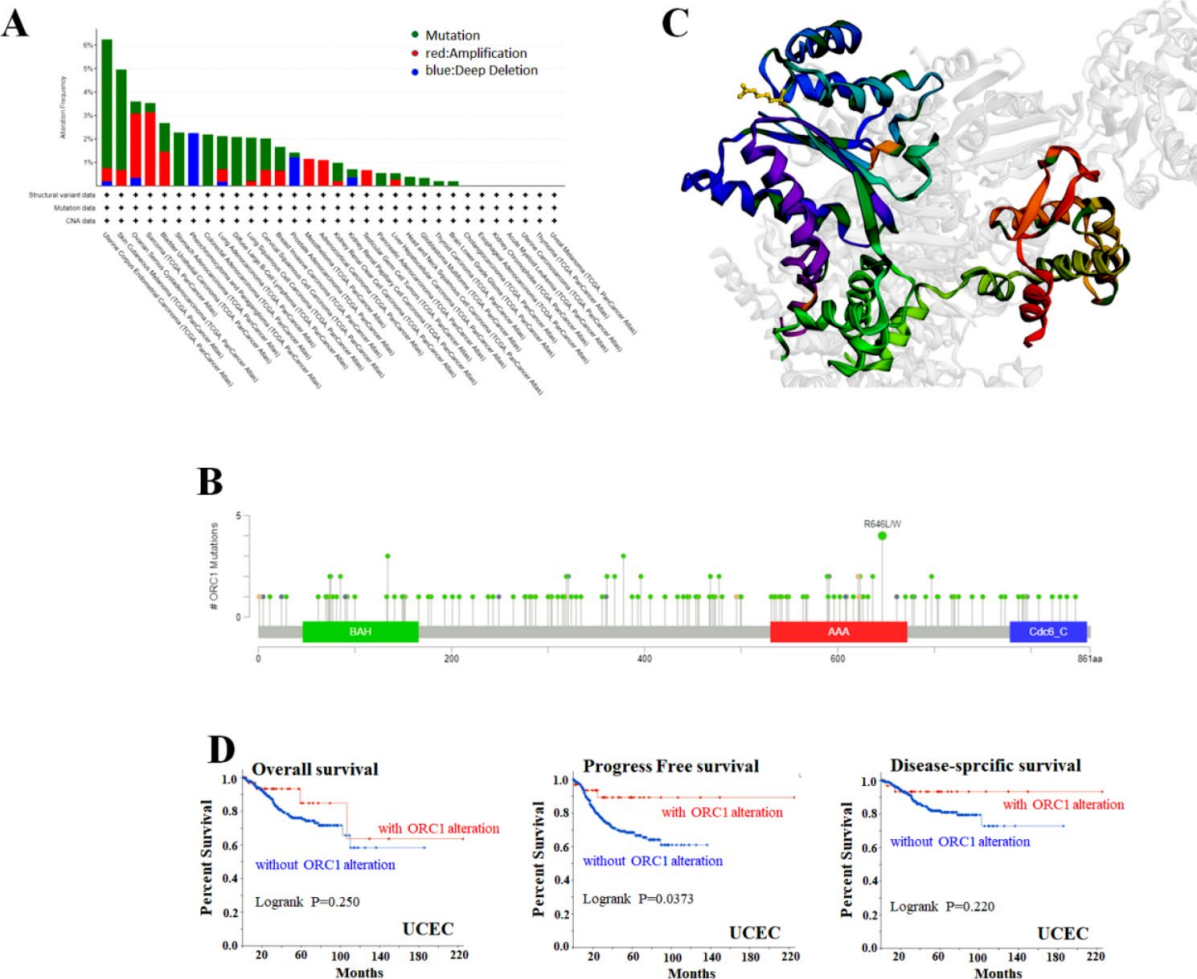
Genetic mutation analysis of ORC1. **A**: The genetic mutation of ORC1 in different tumors. **B**: The mutation sites of ORC1. **C**: The structure of R646L/W which is the highest alteration frequency site. **D**: The potential correlation between ORC1 mutation with the curve of Overall survival, Progress Free survival and Disease-specific survival in normal and tumor tissues

It is well known that the accumulation of genetic mutations is the cause of human cancer [10], so we analyzed which tumor types occurred ORC1 mutation. To explore the mutations of ORC1 in various tumors, we used the TCGA Data-set of the cBioPortal database to analyze the mutation sites and structure of ORC1 in thirty-two kinds of tumors. Results were shown in Fig. 3. ORC1 mutations presented in 25 of the 32 tumors, the mutation frequency of ORC1 in UCEC was the highest among these tumors (Fig. 3A). At the same time, we found that in all the mutation sites of ORC1, R646L/W mutation frequency was the highest, including 1 case of LUAD, 1 case of LUAC, 1 case of RCC and 1 case of COAD (Fig. 3B), so the 3D map of R646LW site in ORC1 protein was also obtained (Fig. 3C). Then the relationship between ORC1 mutation and the prognosis of patients in different tumors was analyzed, it showed the survival of mutant ORC1 was significantly better than that of non-mutant ORC1. In addition, the prognosis of patients in Progress Free survival and disease-specific survival was better than it in Overall survival (Fig. 3D).

### Analysis of ORC1 protein level

**Fig. 4:**
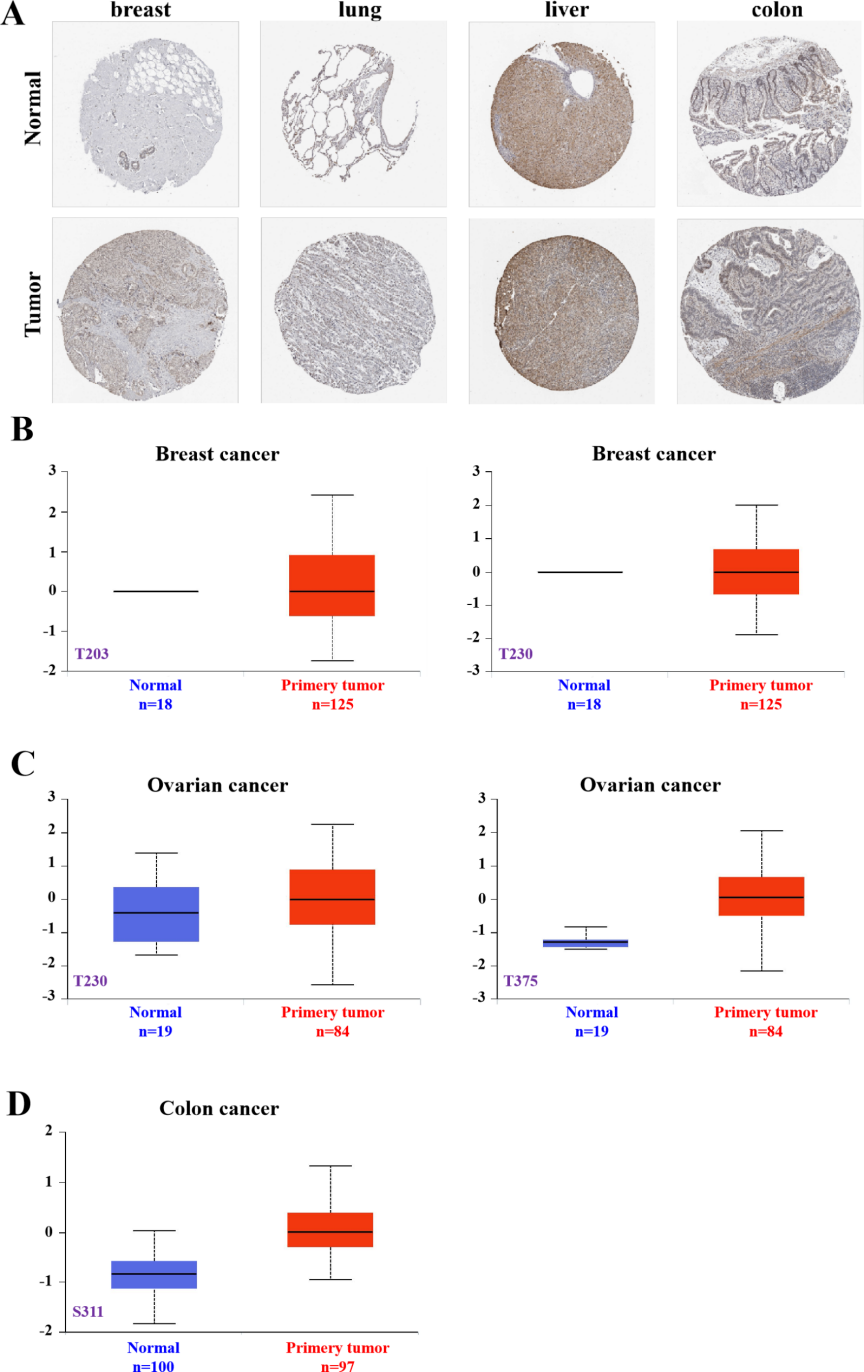
ORC1 protein level analysis. **A**: Based on the HPA database, ORC1 protein expression is shown in normal or neoplastic tissues of the breast, lung, liver, and colon. **B-D**: Phosphorylation levels of ORC1 protein at different loci in breast, ovarian and colon cancers (n = the number of cases)

The protein phosphorylation plays an important role in cell signal transduction, gene expression, cell differentiation and DNA replication in cell cycle [6]. So based on the HPA database, ORC1 protein expression was shown in normal or neoplastic tissues of the breast, lung, liver, and colon(Fig. 4A). It was found that the expression of ORC1 in tumor was higher than normal tissue. Then we performed some analysis of ORC1 protein phosphorylation levels in three tumors, including breast cancer, ovarian cancer and colon cancer. Using the CPTAC Data-set from the UALCAN database, we analyzed the difference of ORC1 phosphorylation levels between normal and tumor tissues at different phosphate sites. The results (Fig. 4B) showed that the protein phosphorylation levels in tumor tissues were significantly higher than those in normal tissues. The phosphorylation levels of T375 in OV and S311 in Colon cancer were significantly increased (Fig. 4C-D).

### The relationship with tumor- infiltrating immune cells

**Fig. 5:**
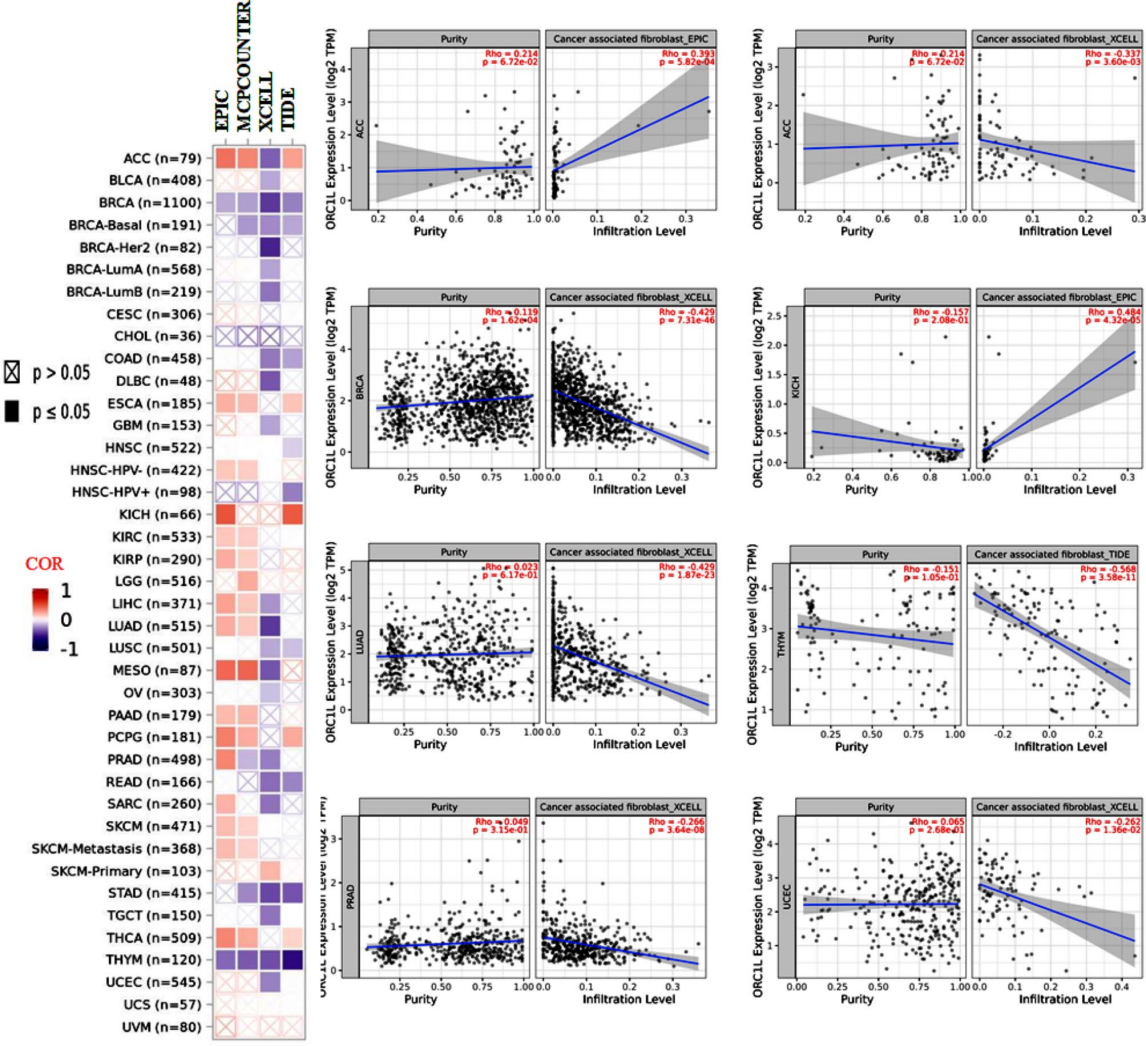
The potential relationship of ORC1 with different levels of immune cell infiltration (n = the number of cases). Relationship between the level of immune cell infiltration and ORC1 expression in tumors. Differential scatter plot of ORC1 expression in tumor tissues with different algorithms

Tumor immunity is an important part of tumor therapy. It mainly studies special immune cells, immune proteins and related signal molecules in tumor micro-environment. In order to study the relationship between ORC1 and the infiltration level of different immune cells in varies tumors, we used EPIC, MCPCOUNTER, XCELL and TIDE algorithms in the TIMER database (Fig. 5), the XCELL and TIDE algorithms estimated the relationship between ORC1 and different levels of immune cell infiltration in 33 tumors. The results showed that ORC1 in ACC and KICH was positively correlated with the infiltration level of immune cells in EPIC. While in ACC, LUAD, BRCA, PAAD and UCEC in the infiltration of XCELL, ORC1 in THYM was negatively correlated with the infiltration level of immune cells.

### ORC1 related genes and proteins

**Fig. 6:**
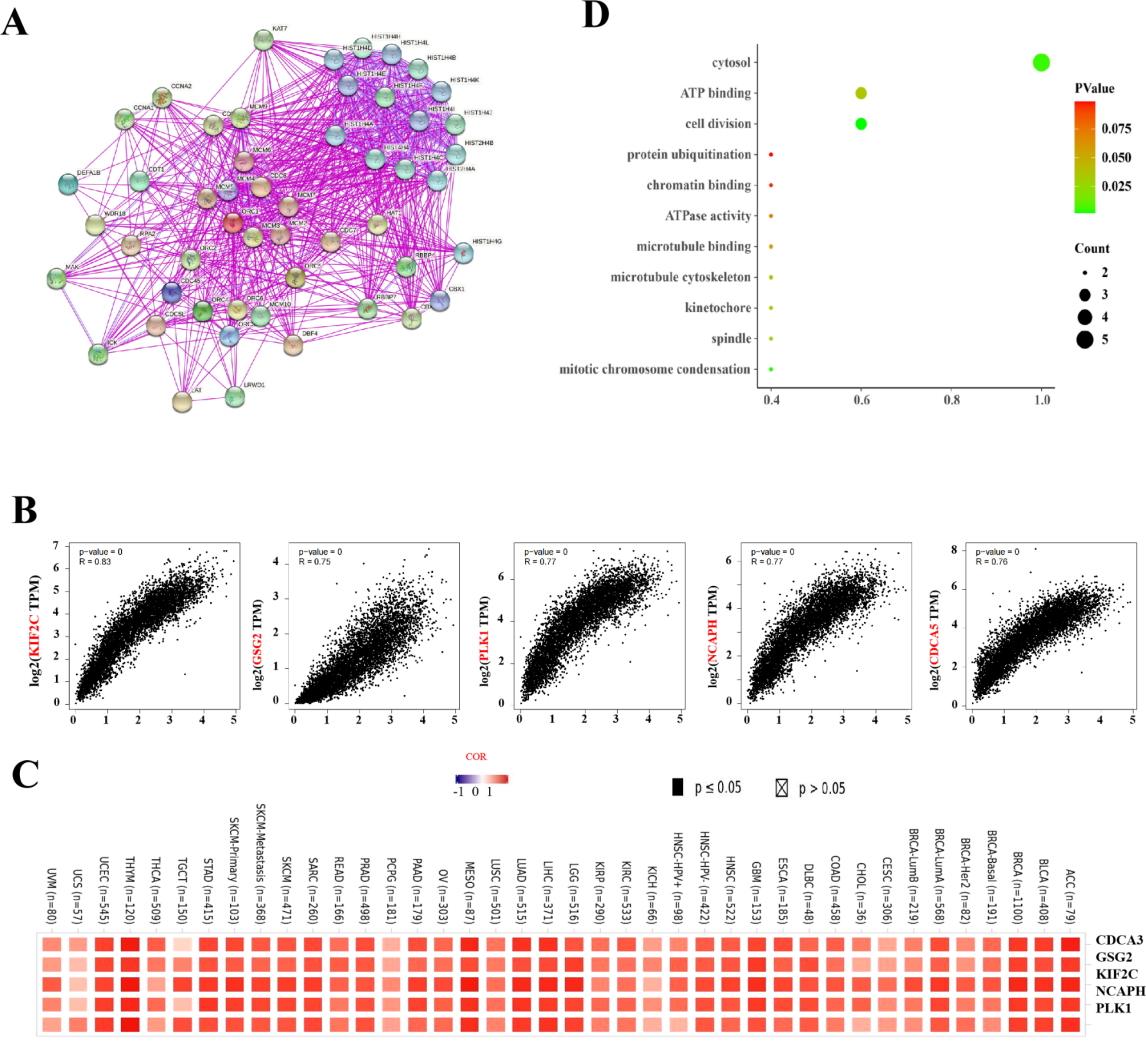
Related Genes and proteins of ORC1. **A**: The co-expression network of ORC1-50 genes related to ORC1 from STRING database. **B**: The correlation between ORC1 expression and the analysis of the first five genes. **C**: The expression of the five genes related to ORC1 and ORC1 expression in different tumors. **D**: KEGG enrichment analysis of ORC1 and ORC1 related genes

In order to further study the molecular mechanism of ORC1 gene in tumorgenesis, we tried to screen the genes related to ORC1 binding protein and ORC1 expression, and study their relationship with different tumors. At first, we got 50 genes related to ORC1 from STRING database (Fig. 6A), and then got 100 genes related to ORC1 expression from GEPIA2, the correlation between ORC1 expression and the analysis of the first five genes (Fig. 6B) and genes associated with ORC1 expression in different tumors were selected. The results showed that CDCA3, GSG2, KIF2C, NCAPH and PLK1 were positively correlated with ORC1, and they were highly correlated with ACC, BLCA, BRCA, LIHC, LUAD, MESO, SKCM, STAD, THYM, UCEC (Fig. 6C). Using KEGG database, the functional enrichment of these genes was analyzed(Fig. 6D). It was found that they are closely related to cytosol, ATP binding and cell division.

### ORC1 expression in tumor tissue was higher than that in adjacent tissue

**Fig. 7:**
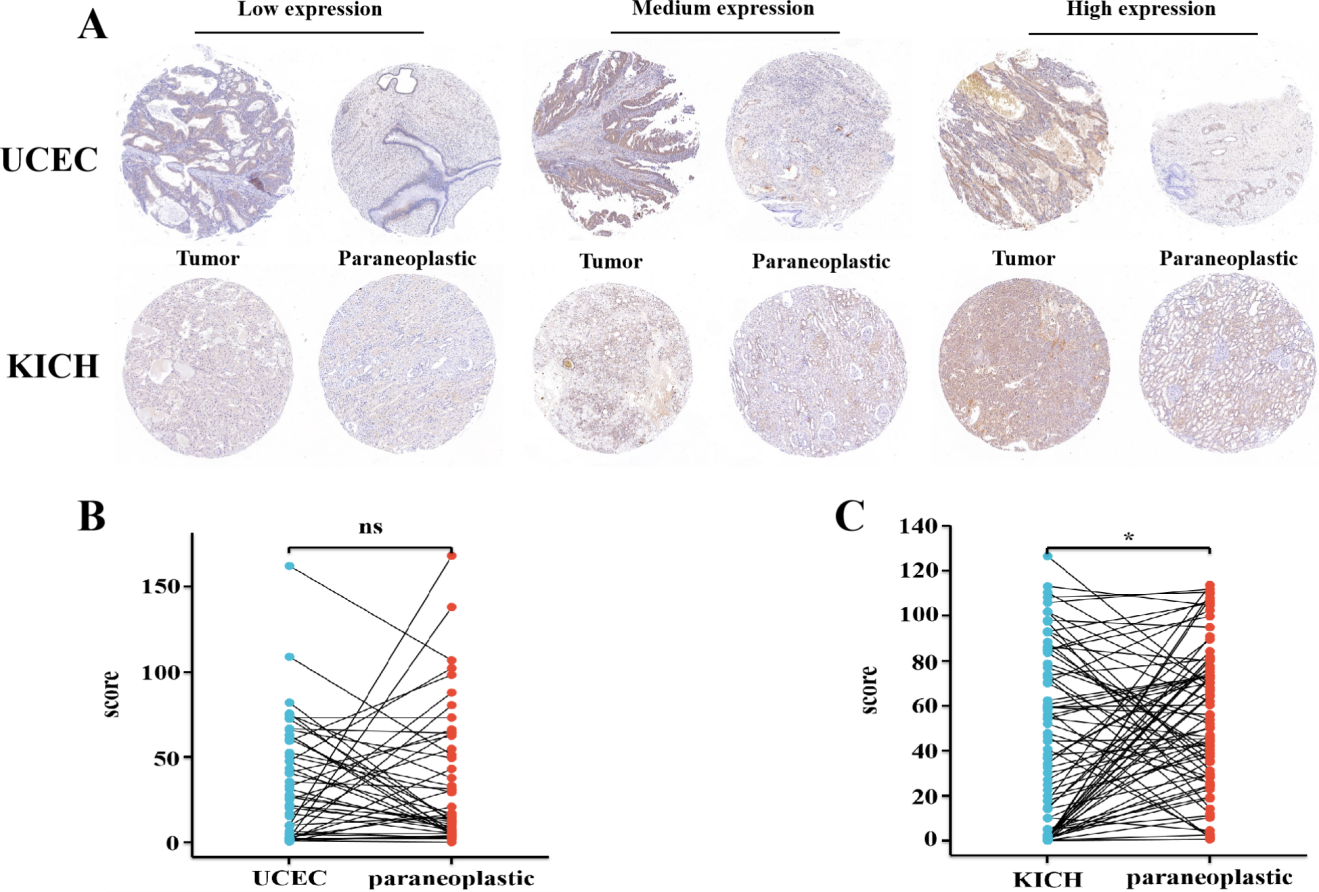
IHC analysis of ORC1 expression. **A**: The expression of ORC1 was analyzed by IHC in UCEC, KICH and their adjacent tissues. **B**: UCEC’s statistical chart (p > 0.05). **C**: KICH’s statistical chart (p < 0.05)

To verify the signature of ORC1 protein in cancer, immunohistochemistry experiments are used in Chromophobe renal cell carcinoma and endometrial cancer tissue microarrays. In Fig. 7A, it is not difficult to find that ORC1 protein is mainly expressed in the nucleus, and there are different expression levels of ORC1 protein in KICC and UCEC cancer tissues. Using H-score statistical immunohistochemistry results, it was found that the expression of ORC1 in KICC was significantly higher than in adjacent tissues (p < 0.05)(Fig. 7C). However, the expression of ORC1 in UCEC was not significantly different (p > 0.05)(Fig. 7B). The results of this trial need to be validated with additional clinical samples.

## Discussion

Cancer is a highly dangerous group of diseases that kills thousands of people every year. The effective treatment of cancer has been a subject of great interest to medical and scientific researchers worldwide. Most studies now recognize that early diagnosis of cancer will help cancer patients receive effective treatment, reduce mortality and improve the quality of life [15, 16]. Finding a biomolecular marker which is specific and broad-spectrum is considered to be an effective and rapid way of diagnosis.

Originating recognition complexes (ORCs) are required to initiate DNA replication in eukaryotic cells and consist of six subunits, ranging from ORC1 to ORC6 [17, 18]. ORC1, also known as HSORC1 in humans, is a key component of the DNA replication licensing machinery, and also plays a role in controlling centromere and centrosome copy number in human cells, independent of its role in DNA replication [19, 20]. ORC1, the largest subunit of ORC, is less expressed in resting cells but can be up-regulated by cell growth signals. The low level of ORC1 expression in quiescent fibroblasts is due to the inhibition of ORC1 promoter transcription in quiescent cells by E2F [21–23]. In rapidly proliferating cells, ORC1 appears highly expressed and localized on chromatin as the cells exit mitosis and form a pre-replicative complex. Later, as cyclin A accumulates and the cell enters S phase, ORC1 is ubiquitinated on chromatin and then degraded. Disruption of ORC1 occurs via the proteasome, signaled in part by the SCF-SKP2 ubiquitin ligase complex [24–26]. ORC1 is ubiquitinated and degraded at the beginning of S phase and then re-synthesized at the end of G2 phase, where it binds to the chromosome as the cell enters mitosis [27, 28]. This differential expression of ORC1 suggests it has the association with the development of cancer.

ORC1 was highly expressed in different types of tumors tissues and matched normal tissues, as detected from the Cancer Genome Atlas (TCGA) and validated by datasets from the gene expression omnibus (GEO) database [29].To explore the relationship between ORC1 expression and clinicopathological features, analysis of the GEPIA2 and UALCAN databases revealed a significant difference in ORC1 expression levels with increasing pathology in ACC, LUAD, OV and SKCM. An analysis of the survival curves of cancer patients found that ORC1 was closely related with poor prognosis in ACC, LIHC, PAAD, READ and THCA. ORC1 in ACC and KICH. Phosphorylation levels of T375 in OV and S311 in colon cancer ORC1 protein were found to be significantly increased by using the CPTAC dataset from the UALCAN database. Application of multiple immune deconvolution methods, we found ORC1 was positively correlated with the infiltration level of immune cells while in THYM ORC1 was negatively correlated with the infiltration level of immune cells. Co-expression network analysis showed that CDCA3, GSG2, KIF2C, NCAPH and PLK1 were positively correlated with ORC1 in cancer, and the functional enrichment mainly included cytosol, ATP binding and cell division. Meanwhile, we verified the difference of ORC1 expression between tumor tissues and adjacent tissues by immunohistochemistry, and found that ORC1 expression was different between tumor tissues and paracancerous tissues, the expression in both UCEC and KICH were higher than adjacent tissue. These results showed ORC1 over-expressed in most tumors and could be severed as a novel biomarker for diagnosis. Meanwhile it revealed that ORC1 might inhibit the tumor immunity and might be a potential therapeutic target in cancers.

### Electronic supplementary material

Below is the link to the electronic supplementary material.

Supplementary Material 1

## Data Availability

The results were analysed online and aggregated directly from multiple databases without relevant accession numbers. Direct web links of datasets: GEPIA2, http://gepia.cancer-pku.cn/version 2; UALCAN, http://ualcan.path.uab.edu/; cBioPortal, https://www.cbioportal.org/; Human Protein Atlas, https://www.proteinatlas.org/; The TIMER database, http://cistrome.dfci.harvard.edu/TIMER/; STRING, https://cn.string-db.org/.
