# Supplementary material for: Origin recognition complex subunit 1(ORC1) is a potential biomarker and therapeutic target in cancer
Source: BMC Med Genomics. 2023 Oct 13;16:243. doi: 10.1186/s12920-023-01691-9 (PMC10571394; doi:10.1186/s12920-023-01691-9)
Supplement: Supplementary file 1 — Supplementary Material 1 [file 12920_2023_1691_MOESM1_ESM.docx]

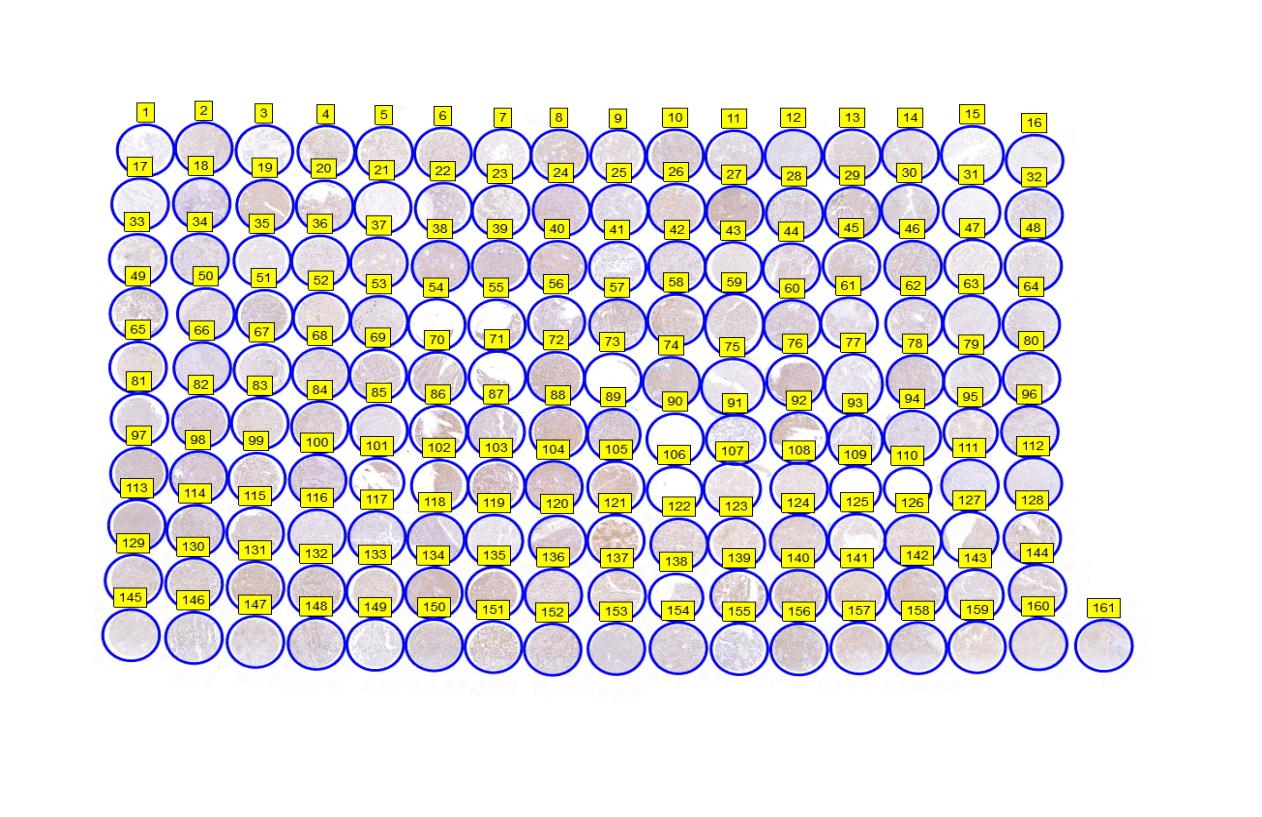


**Figure S1.** The immunohistochemistry of ORC1 expression in human KICC microarray ZL-KICC1601.

**Table S1.** The Immunohistochemical analysis results of human KICC microarray ZL-KICC1601.

| **DensitoQuant** | **No. Negative pixels** | **No. Weak-Positive pixels** | **No. Moderate-Positive pixels** | **No. Strong-Positive pixels** | **H-Score** |
| --- | --- | --- | --- | --- | --- |
| 1 | 4693839.00 | 2535.00 | 2227.00 | 673.00 | 0.19 |
| 2 | 3688114.00 | 2495074.00 | 646763.00 | 8216.00 | 55.76 |
| 3 | 2277618.00 | 54349.00 | 2470.00 | 1389.00 | 2.72 |
| 4 | 2544301.00 | 2387924.00 | 1307399.00 | 36771.00 | 81.46 |
| 5 | 2287999.00 | 2263892.00 | 134958.00 | 1749.00 | 54.15 |
| 6 | 2903014.00 | 2350143.00 | 1040440.00 | 81681.00 | 73.35 |
| 7 | 1828193.00 | 247654.00 | 17380.00 | 5102.00 | 14.19 |
| 8 | 2957066.00 | 4726455.00 | 1477188.00 | 12178.00 | 84.13 |
| 9 | 1636509.00 | 803817.00 | 54632.00 | 656.00 | 36.67 |
| 10 | 3903111.00 | 3383679.00 | 1719853.00 | 23817.00 | 76.35 |
| 11 | 3397779.00 | 304665.00 | 32861.00 | 3101.00 | 10.16 |
| 12 | 5898565.00 | 256370.00 | 7436.00 | 2116.00 | 4.50 |
| 13 | 2463462.00 | 1502644.00 | 5916.00 | 411.00 | 38.16 |
| 14 | 3029703.00 | 2078487.00 | 57250.00 | 551.00 | 42.48 |
| 15 | 3392750.00 | 398.00 | 112.00 | 59.00 | 0.02 |
| 16 | 4176714.00 | 24597.00 | 1556.00 | 204.00 | 0.67 |
| 17 | 4415493.00 | 8791.00 | 2146.00 | 119.00 | 0.30 |
| 18 | 10300754.00 | 222423.00 | 19101.00 | 2954.00 | 2.56 |
| 19 | 1931980.00 | 8756568.00 | 1100705.00 | 3874.00 | 93.02 |
| 20 | 2763182.00 | 2305474.00 | 565166.00 | 20921.00 | 61.87 |
| 21 | 2749041.00 | 3891.00 | 2666.00 | 4555.00 | 0.83 |
| 22 | 3941900.00 | 1005316.00 | 261735.00 | 4199.00 | 29.57 |
| 23 | 2604419.00 | 1283755.00 | 264239.00 | 9462.00 | 44.23 |
| 24 | 6350535.00 | 2931178.00 | 788521.00 | 18789.00 | 45.24 |
| 25 | 5677213.00 | 41267.00 | 2508.00 | 1682.00 | 0.90 |
| 26 | 2544085.00 | 5251633.00 | 937174.00 | 2969.00 | 81.67 |
| 27 | 2724570.00 | 8034262.00 | 1737937.00 | 25800.00 | 92.53 |
| 28 | 4619935.00 | 2019594.00 | 193126.00 | 198.00 | 35.22 |
| 29 | 2812964.00 | 3609664.00 | 803896.00 | 55264.00 | 73.93 |
| 30 | 4744331.00 | 1175396.00 | 166409.00 | 596.00 | 24.81 |
| 31 | 3067384.00 | 25951.00 | 9324.00 | 2561.00 | 1.68 |
| 32 | 4619120.00 | 772722.00 | 237445.00 | 12992.00 | 22.80 |
| 33 | 2234451.00 | 1304183.00 | 193240.00 | 36148.00 | 47.75 |
| 34 | 5593216.00 | 1015066.00 | 386670.00 | 3120.00 | 25.69 |
| 35 | 3642940.00 | 15068.00 | 738.00 | 123.00 | 0.46 |
| 36 | 3786802.00 | 1285303.00 | 290522.00 | 3075.00 | 34.95 |
| 37 | 2492878.00 | 809894.00 | 46813.00 | 1092.00 | 27.06 |
| 38 | 4832637.00 | 3977982.00 | 1352671.00 | 24390.00 | 66.32 |
| 39 | 4262598.00 | 2116388.00 | 137927.00 | 139.00 | 36.71 |
| 40 | 4140015.00 | 3829102.00 | 1637700.00 | 40681.00 | 74.91 |
| 41 | 6026381.00 | 30489.00 | 4844.00 | 847.00 | 0.70 |
| 42 | 4694911.00 | 1325360.00 | 70794.00 | 333.00 | 24.10 |
| 43 | 2226121.00 | 649913.00 | 31109.00 | 3092.00 | 24.79 |
| 44 | 3747894.00 | 2499101.00 | 427278.00 | 2400.00 | 50.34 |
| 45 | 3150471.00 | 3395621.00 | 339104.00 | 170.00 | 59.17 |
| 46 | 4690225.00 | 2854117.00 | 951241.00 | 3572.00 | 56.09 |
| 47 | 2856326.00 | 126119.00 | 8488.00 | 913.00 | 4.87 |
| 48 | 3459398.00 | 758827.00 | 31704.00 | 274.00 | 19.37 |
| 49 | 2171655.00 | 1917785.00 | 1286013.00 | 60188.00 | 85.92 |
| 50 | 4526585.00 | 1161533.00 | 610393.00 | 11030.00 | 38.28 |
| 51 | 3266204.00 | 2546228.00 | 647092.00 | 1762.00 | 59.52 |
| 52 | 1783569.00 | 1929242.00 | 190141.00 | 17535.00 | 60.25 |
| 53 | 3452538.00 | 732264.00 | 130156.00 | 180952.00 | 34.15 |
| 54 | 475305.00 | 275358.00 | 341178.00 | 8874.00 | 89.43 |
| 55 | 1435594.00 | 1788127.00 | 932980.00 | 8425.00 | 88.34 |
| 56 | 4858273.00 | 1135127.00 | 308709.00 | 10915.00 | 28.28 |
| 57 | 3335906.00 | 3012282.00 | 1093510.00 | 18811.00 | 70.45 |
| 58 | 2389024.00 | 4411825.00 | 2465102.00 | 74395.00 | 102.41 |
| 59 | 2288055.00 | 1741412.00 | 311528.00 | 416.00 | 54.49 |
| 60 | 5042001.00 | 2579140.00 | 573408.00 | 20560.00 | 46.11 |
| 61 | 4530254.00 | 373373.00 | 55180.00 | 3442.00 | 9.96 |
| 62 | 3950175.00 | 1575590.00 | 447502.00 | 8036.00 | 41.71 |
| 63 | 4046951.00 | 45703.00 | 2254.00 | 307.00 | 1.25 |
| 64 | 5105232.00 | 602268.00 | 242646.00 | 9205.00 | 18.71 |
| 65 | 1689440.00 | 2068712.00 | 72140.00 | 3294.00 | 57.98 |
| 66 | 9016048.00 | 121658.00 | 963.00 | 129.00 | 1.36 |
| 67 | 3761480.00 | 416873.00 | 89773.00 | 1891.00 | 14.10 |
| 68 | 4119129.00 | 1065907.00 | 254529.00 | 6610.00 | 29.28 |
| 69 | 2868927.00 | 1672829.00 | 581727.00 | 10267.00 | 55.85 |
| 70 | 3261625.00 | 2872464.00 | 1045446.00 | 27226.00 | 70.00 |
| 71 | 880769.00 | 633090.00 | 522593.00 | 21569.00 | 84.69 |
| 72 | 3654201.00 | 4329257.00 | 3460417.00 | 79539.00 | 99.70 |
| 73 | 449506.00 | 3910.00 | 486.00 | 43.00 | 1.10 |
| 74 | 4434766.00 | 4159544.00 | 1541044.00 | 65480.00 | 72.92 |
| 75 | 4363625.00 | 29517.00 | 9874.00 | 2269.00 | 1.27 |
| 76 | 3093233.00 | 4372066.00 | 2124730.00 | 40445.00 | 90.78 |
| 77 | 4821435.00 | 14671.00 | 2653.00 | 630.00 | 0.45 |
| 78 | 4207659.00 | 3423496.00 | 1055681.00 | 15790.00 | 64.14 |
| 79 | 3953680.00 | 190008.00 | 9462.00 | 2297.00 | 5.19 |
| 80 | 3822180.00 | 870639.00 | 149543.00 | 4289.00 | 24.40 |
| 81 | 3796375.00 | 79984.00 | 6810.00 | 490.00 | 2.45 |
| 82 | 5491547.00 | 2407676.00 | 822120.00 | 19392.00 | 47.02 |
| 83 | 1968760.00 | 2327805.00 | 192547.00 | 1269.00 | 60.50 |
| 84 | 3046610.00 | 2694800.00 | 1163831.00 | 14581.00 | 73.21 |
| 85 | 4335960.00 | 36315.00 | 4932.00 | 170.00 | 1.07 |
| 86 | 2272862.00 | 2674387.00 | 2579872.00 | 146495.00 | 107.82 |
| 87 | 3646367.00 | 582257.00 | 72010.00 | 10680.00 | 17.59 |
| 88 | 2388572.00 | 5708645.00 | 3854585.00 | 84722.00 | 113.59 |
| 89 | 3707352.00 | 2079176.00 | 300708.00 | 53365.00 | 46.26 |
| 90 | 65801.00 | 229.00 | 261.00 | 116.00 | 1.65 |
| 91 | 5927229.00 | 4215.00 | 907.00 | 399.00 | 0.12 |
| 92 | 1239970.00 | 2614088.00 | 1647115.00 | 67881.00 | 109.75 |
| 93 | 3425963.00 | 138347.00 | 13208.00 | 5399.00 | 5.05 |
| 94 | 6134546.00 | 543854.00 | 99837.00 | 2797.00 | 11.09 |
| 95 | 2053361.00 | 2118466.00 | 31670.00 | 246.00 | 51.92 |
| 96 | 4538539.00 | 1309231.00 | 193895.00 | 986.00 | 28.13 |
| 97 | 3534353.00 | 3834002.00 | 1606405.00 | 5504.00 | 78.65 |
| 98 | 4903809.00 | 2550187.00 | 937254.00 | 33522.00 | 53.71 |
| 99 | 2275498.00 | 3180118.00 | 394636.00 | 52381.00 | 69.91 |
| 100 | 6596162.00 | 1635104.00 | 740130.00 | 29121.00 | 35.58 |
| 101 | 640554.00 | 957157.00 | 775064.00 | 30865.00 | 108.16 |
| 102 | 1637543.00 | 2711263.00 | 2134282.00 | 93534.00 | 110.40 |
| 103 | 2292515.00 | 5731931.00 | 1585222.00 | 13279.00 | 92.93 |
| 104 | 2557457.00 | 5916995.00 | 3021242.00 | 83369.00 | 105.45 |
| 105 | 1813891.00 | 8682871.00 | 1469210.00 | 15250.00 | 97.38 |
| 106 | 74241.00 | 6487.00 | 2168.00 | 344.00 | 14.24 |
| 107 | 1805002.00 | 620738.00 | 63339.00 | 476.00 | 30.08 |
| 108 | 2816533.00 | 1562806.00 | 227833.00 | 8660.00 | 44.29 |
| 109 | 51335.00 | 26687.00 | 3048.00 | 70.00 | 40.66 |
| 110 | 13941.00 | 303.00 | 40.00 | 4.00 | 2.76 |
| 111 | 8987291.00 | 1999.00 | 766.00 | 281.00 | 0.05 |
| 112 | 6057299.00 | 35706.00 | 343.00 | 72.00 | 0.60 |
| 113 | 3634380.00 | 4754056.00 | 112148.00 | 1163.00 | 58.60 |
| 114 | 4224165.00 | 2665504.00 | 1750866.00 | 56205.00 | 72.85 |
| 115 | 2526738.00 | 2887029.00 | 813707.00 | 637.00 | 72.52 |
| 116 | 4895005.00 | 552692.00 | 41840.00 | 7560.00 | 11.99 |
| 117 | 6594339.00 | 7981.00 | 1714.00 | 273.00 | 0.19 |
| 118 | 5225905.00 | 567310.00 | 11958.00 | 308.00 | 10.20 |
| 119 | 3451129.00 | 68394.00 | 6701.00 | 824.00 | 2.39 |
| 120 | 3638634.00 | 3155599.00 | 883333.00 | 16846.00 | 64.63 |
| 121 | 570179.00 | 7890776.00 | 3763102.00 | 20482.00 | 126.41 |
| 122 | 3210797.00 | 3695598.00 | 756953.00 | 7500.00 | 68.21 |
| 123 | 1359154.00 | 3938789.00 | 538151.00 | 5014.00 | 86.12 |
| 124 | 2689006.00 | 4376511.00 | 1058703.00 | 2227.00 | 79.99 |
| 125 | 1471623.00 | 219133.00 | 14926.00 | 998.00 | 14.76 |
| 126 | 2697533.00 | 2161768.00 | 1291366.00 | 9617.00 | 77.49 |
| 127 | 1475495.00 | 3640213.00 | 94268.00 | 416.00 | 73.51 |
| 128 | 2668615.00 | 3615140.00 | 1203998.00 | 3506.00 | 80.54 |
| 129 | 2705587.00 | 3868032.00 | 152499.00 | 7862.00 | 62.32 |
| 130 | 3012352.00 | 3997064.00 | 863003.00 | 12228.00 | 73.05 |
| 131 | 1654038.00 | 7931106.00 | 3255131.00 | 38116.00 | 113.02 |
| 132 | 2690551.00 | 2898296.00 | 2760088.00 | 161361.00 | 104.61 |
| 133 | 1550590.00 | 2653679.00 | 466430.00 | 172698.00 | 84.75 |
| 134 | 2666829.00 | 6255292.00 | 3366907.00 | 53855.00 | 106.54 |
| 135 | 1702968.00 | 6518379.00 | 2807661.00 | 16032.00 | 110.29 |
| 136 | 3430546.00 | 2949111.00 | 1157337.00 | 9351.00 | 70.12 |
| 137 | 1353477.00 | 4884958.00 | 1271918.00 | 107732.00 | 101.76 |
| 138 | 2505737.00 | 776529.00 | 143277.00 | 1305.00 | 31.14 |
| 139 | 1821592.00 | 3142414.00 | 2148599.00 | 43616.00 | 105.79 |
| 140 | 2097139.00 | 3545902.00 | 3048574.00 | 37276.00 | 111.75 |
| 141 | 990304.00 | 5728804.00 | 837693.00 | 477.00 | 97.99 |
| 142 | 2944571.00 | 5209893.00 | 2362699.00 | 18790.00 | 94.83 |
| 143 | 2338332.00 | 656890.00 | 8887.00 | 440.00 | 22.50 |
| 144 | 3771819.00 | 2346243.00 | 219964.00 | 351.00 | 43.97 |
| 145 | 2689068.00 | 558189.00 | 11232.00 | 97.00 | 17.83 |
| 146 | 4352755.00 | 1260047.00 | 560668.00 | 16125.00 | 39.26 |
| 147 | 2803140.00 | 1073793.00 | 108862.00 | 1523.00 | 32.51 |
| 148 | 4409085.00 | 1582306.00 | 894860.00 | 29152.00 | 50.03 |
| 149 | 4297991.00 | 86558.00 | 9920.00 | 419.00 | 2.45 |
| 150 | 3984807.00 | 2312850.00 | 1620606.00 | 63094.00 | 71.96 |
| 151 | 2087443.00 | 2827355.00 | 2138236.00 | 32607.00 | 101.64 |
| 152 | 4114899.00 | 2008593.00 | 1507872.00 | 45743.00 | 67.23 |
| 153 | 5309871.00 | 973852.00 | 149481.00 | 2838.00 | 19.91 |
| 154 | 4669836.00 | 1713964.00 | 413264.00 | 368.00 | 37.39 |
| 155 | 5933986.00 | 39289.00 | 14083.00 | 5222.00 | 1.39 |
| 156 | 3555173.00 | 4087528.00 | 1706694.00 | 6403.00 | 80.38 |
| 157 | 1809409.00 | 4713416.00 | 243822.00 | 1050.00 | 76.90 |
| 158 | 3776857.00 | 2062803.00 | 620130.00 | 19678.00 | 51.89 |
| 159 | 1221896.00 | 3417489.00 | 386763.00 | 3082.00 | 83.52 |
| 160 | 3278096.00 | 1816510.00 | 149404.00 | 1644.00 | 40.42 |
| 161 | 4748044.00 | 723454.00 | 13725.00 | 437.00 | 13.71 |

**Figure S2.** The immunohistochemistry of ORC1 expression in endometrial cancer tissue microarray ZL-UteS961.


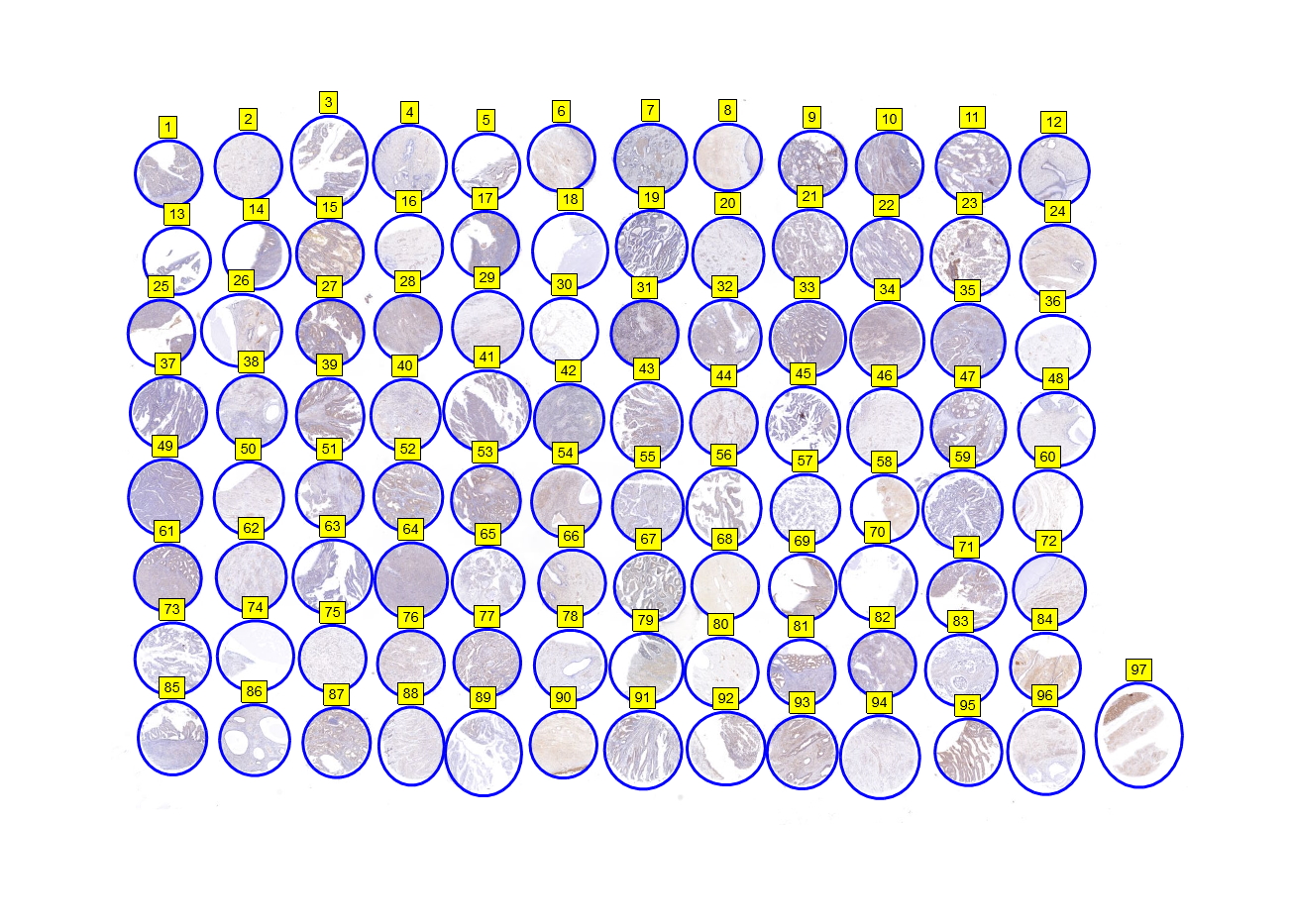


**Table S2.** The Immunohistochemical analysis results of human endometrial cancer tissue microarray ZL-UteS961.

| **DensitoQuant** | **No. Negative pixels** | **No. Weak-Positive pixels** | **No. Moderate-Positive pixels** | **No. Strong-Positive pixels** | **H-Score** |
| --- | --- | --- | --- | --- | --- |
| 1 | 8288894.00 | 852163.00 | 219425.00 | 727.00 | 13.81 |
| 2 | 3775011.00 | 285823.00 | 23057.00 | 782.00 | 8.18 |
| 3 | 4303810.00 | 451708.00 | 240525.00 | 2414.00 | 18.81 |
| 4 | 6367769.00 | 110829.00 | 48523.00 | 3245.00 | 3.33 |
| 5 | 2934747.00 | 180614.00 | 247539.00 | 19436.00 | 21.70 |
| 6 | 3176569.00 | 1471596.00 | 237687.00 | 22363.00 | 41.03 |
| 7 | 11221471.00 | 575428.00 | 330231.00 | 2128.00 | 10.24 |
| 8 | 2242933.00 | 1287231.00 | 33877.00 | 2058.00 | 38.17 |
| 9 | 8921965.00 | 885039.00 | 822549.00 | 159763.00 | 27.89 |
| 10 | 11180863.00 | 1860904.00 | 480467.00 | 1630.00 | 20.90 |
| 11 | 10865583.00 | 357960.00 | 179119.00 | 7392.00 | 6.47 |
| 12 | 8992058.00 | 95904.00 | 38267.00 | 4313.00 | 2.03 |
| 13 | 3631650.00 | 26508.00 | 10862.00 | 124.00 | 1.32 |
| 14 | 6694190.00 | 206107.00 | 175762.00 | 1540.00 | 7.94 |
| 15 | 2772510.00 | 4337317.00 | 4526711.00 | 60713.00 | 116.03 |
| 16 | 3403916.00 | 187323.00 | 24493.00 | 1188.00 | 6.63 |
| 17 | 9042842.00 | 1152384.00 | 734157.00 | 5421.00 | 24.12 |
| 18 | 10330839.00 | 4177.00 | 3110.00 | 667.00 | 0.12 |
| 19 | 12937057.00 | 92573.00 | 189374.00 | 14658.00 | 3.89 |
| 20 | 5430082.00 | 181767.00 | 58187.00 | 3666.00 | 5.45 |
| 21 | 7931963.00 | 640828.00 | 142589.00 | 3202.00 | 10.73 |
| 22 | 10400376.00 | 240488.00 | 69432.00 | 4738.00 | 3.67 |
| 23 | 4400508.00 | 742538.00 | 1461706.00 | 446110.00 | 70.97 |
| 24 | 2193508.00 | 1837874.00 | 233999.00 | 19725.00 | 55.19 |
| 25 | 5154218.00 | 650555.00 | 842844.00 | 37796.00 | 36.64 |
| 26 | 4811167.00 | 703766.00 | 242764.00 | 28730.00 | 22.04 |
| 27 | 7876793.00 | 1408943.00 | 3869002.00 | 137733.00 | 71.92 |
| 28 | 7535336.00 | 2798291.00 | 567984.00 | 6180.00 | 36.24 |
| 29 | 4774520.00 | 351663.00 | 112623.00 | 1579.00 | 11.10 |
| 30 | 1656068.00 | 48100.00 | 10249.00 | 840.00 | 4.15 |
| 31 | 11414558.00 | 1527897.00 | 1144695.00 | 108473.00 | 29.18 |
| 32 | 7680151.00 | 1053520.00 | 164731.00 | 1468.00 | 15.59 |
| 33 | 9403105.00 | 984694.00 | 920390.00 | 10269.00 | 25.24 |
| 34 | 6460930.00 | 1873520.00 | 1146241.00 | 24381.00 | 44.60 |
| 35 | 11001906.00 | 825437.00 | 216513.00 | 11073.00 | 10.71 |
| 36 | 1886964.00 | 47277.00 | 28537.00 | 3603.00 | 5.86 |
| 37 | 12687307.00 | 121618.00 | 27403.00 | 1219.00 | 1.40 |
| 38 | 8876465.00 | 205643.00 | 29776.00 | 2180.00 | 2.98 |
| 39 | 5868476.00 | 1301073.00 | 1885987.00 | 36705.00 | 57.01 |
| 40 | 5619149.00 | 428888.00 | 139628.00 | 16098.00 | 12.19 |
| 41 | 6786463.00 | 441514.00 | 322611.00 | 13927.00 | 14.92 |
| 42 | 12528758.00 | 740133.00 | 94627.00 | 19376.00 | 7.38 |
| 43 | 6262657.00 | 1175860.00 | 439070.00 | 1576.00 | 26.13 |
| 44 | 3994251.00 | 1151756.00 | 424046.00 | 2307.00 | 36.01 |
| 45 | 6527516.00 | 140658.00 | 33915.00 | 38361.00 | 4.80 |
| 46 | 3504516.00 | 136744.00 | 8852.00 | 85.00 | 4.24 |
| 47 | 10010471.00 | 907376.00 | 173186.00 | 583.00 | 11.32 |
| 48 | 5069169.00 | 54114.00 | 20324.00 | 2603.00 | 1.99 |
| 49 | 16846918.00 | 196316.00 | 52853.00 | 1881.00 | 1.80 |
| 50 | 3767935.00 | 355016.00 | 85416.00 | 1201.00 | 12.58 |
| 51 | 9668125.00 | 467651.00 | 299651.00 | 11439.00 | 10.54 |
| 52 | 7830131.00 | 1849974.00 | 1515028.00 | 25643.00 | 44.18 |
| 53 | 8078233.00 | 2030351.00 | 1968863.00 | 21717.00 | 49.86 |
| 54 | 5576157.00 | 2556511.00 | 303429.00 | 1397.00 | 37.54 |
| 55 | 9856406.00 | 264543.00 | 44870.00 | 887.00 | 3.51 |
| 56 | 3216973.00 | 736247.00 | 187420.00 | 5187.00 | 27.18 |
| 57 | 6357435.00 | 19339.00 | 3179.00 | 665.00 | 0.43 |
| 58 | 644226.00 | 2320374.00 | 150577.00 | 8690.00 | 84.75 |
| 59 | 11082383.00 | 198610.00 | 16458.00 | 1831.00 | 2.10 |
| 60 | 1322565.00 | 380073.00 | 117385.00 | 24426.00 | 37.31 |
| 61 | 10193742.00 | 1565828.00 | 951224.00 | 17689.00 | 27.67 |
| 62 | 7325662.00 | 414170.00 | 86165.00 | 17773.00 | 8.16 |
| 63 | 9145792.00 | 51600.00 | 46376.00 | 4201.00 | 1.70 |
| 64 | 10979869.00 | 1873431.00 | 177102.00 | 4194.00 | 17.19 |
| 65 | 6609872.00 | 120677.00 | 28133.00 | 6276.00 | 2.89 |
| 66 | 4529698.00 | 1088671.00 | 130334.00 | 14180.00 | 24.15 |
| 67 | 7369660.00 | 1772584.00 | 1122409.00 | 24977.00 | 39.77 |
| 68 | 3010362.00 | 1169174.00 | 47913.00 | 3910.00 | 30.17 |
| 69 | 3118187.00 | 1426914.00 | 1546449.00 | 160304.00 | 79.99 |
| 70 | 2944188.00 | 12152.00 | 1606.00 | 238.00 | 0.54 |
| 71 | 4524631.00 | 1251513.00 | 852827.00 | 46001.00 | 46.37 |
| 72 | 8350234.00 | 1272809.00 | 455551.00 | 4883.00 | 21.80 |
| 73 | 7867739.00 | 28413.00 | 2949.00 | 2431.00 | 0.53 |
| 74 | 3148061.00 | 3812.00 | 305.00 | 26.00 | 0.14 |
| 75 | 4339607.00 | 224198.00 | 39645.00 | 2425.00 | 6.75 |
| 76 | 5248617.00 | 1047689.00 | 102189.00 | 1832.00 | 19.65 |
| 77 | 7399658.00 | 1219859.00 | 943554.00 | 21060.00 | 33.08 |
| 78 | 5136991.00 | 174744.00 | 10619.00 | 383.00 | 3.70 |
| 79 | 5541769.00 | 2378103.00 | 473036.00 | 7008.00 | 39.82 |
| 80 | 2030189.00 | 141960.00 | 73853.00 | 36322.00 | 17.47 |
| 81 | 9391605.00 | 848348.00 | 1249720.00 | 41013.00 | 30.10 |
| 82 | 11175767.00 | 1232296.00 | 173239.00 | 9813.00 | 12.77 |
| 83 | 8112534.00 | 60876.00 | 5190.00 | 537.00 | 0.89 |
| 84 | 1281340.00 | 4033729.00 | 1547510.00 | 131105.00 | 107.56 |
| 85 | 9251684.00 | 239062.00 | 93450.00 | 3574.00 | 4.55 |
| 86 | 6855629.00 | 73524.00 | 38313.00 | 11419.00 | 2.64 |
| 87 | 6060735.00 | 2186694.00 | 899782.00 | 7863.00 | 43.80 |
| 88 | 4291518.00 | 193447.00 | 22746.00 | 2470.00 | 5.46 |
| 89 | 7619351.00 | 62030.00 | 11626.00 | 1311.00 | 1.16 |
| 90 | 1022017.00 | 2596052.00 | 376721.00 | 198196.00 | 94.06 |
| 91 | 6813592.00 | 791006.00 | 444997.00 | 5104.00 | 21.06 |
| 92 | 2662137.00 | 916651.00 | 570138.00 | 32656.00 | 51.53 |
| 93 | 4788186.00 | 1927755.00 | 1944618.00 | 118840.00 | 70.32 |
| 94 | 4642428.00 | 220170.00 | 29107.00 | 1350.00 | 5.77 |
| 95 | 2321495.00 | 2077850.00 | 3015894.00 | 78450.00 | 111.36 |
| 96 | 3486233.00 | 644416.00 | 94191.00 | 2623.00 | 19.89 |
| 97 | 1010291.00 | 1266196.00 | 1182560.00 | 117716.00 | 111.40 |
